# A risk profile of compulsive exercise in adolescents with an eating disorder, and their affective responses to an inpatient exercise program

**DOI:** 10.1186/2050-2974-3-S1-O47

**Published:** 2015-11-23

**Authors:** Melissa Fietz, Phillipa Hay, Jane Miskovic-Wheatley, Sloane Madden, Stephen Touyz

**Affiliations:** 1 University of Sydney, Sydney, Australia; 2 University of Western Sydney, Sydney, Australia; 3 Children's Hospital at Westmead, Sydney, Australia

## 

Compulsive exercise is known to be a problematic feature of adult eating disorders (ED), however, there is little research investigating compulsive exercise in adolescents with EDs. In particular, the acute anxiolytic and anti-depressant properties of exercise are yet to be determined in adolescent ED inpatients. Thus, the current study examined the prevalence and psychopathological correlates of compulsive exercise in a sample of 60 female adolescent ED inpatients. The study also aimed to identify the psychological benefits of a supervised inpatient physiotherapy-lead exercise program, and if these benefits were greater in participants reporting higher levels of compulsive exercise. At the commencement of their inpatient treatment participants completed a series of standardised self-report questionnaires, assessing eating and psychiatric pathology as well as compulsive exercise features. Participants' mood state was then repeatedly assessed immediately before and after participating in physiotherapy sessions (exercise condition) and school classes (control condition) using visual analogue scales for an admission period of up to three weeks. Results indicated that higher levels of ED symptomology and anxiety were significantly associated with compulsive exercise. Significant acute mood benefits in response to the exercise intervention were also identified in preliminary analyses. The findings support the relevance of assessing compulsive exercise in adolescents with EDs and provide an understanding of the positive and negative reinforcement functions of exercise, which may guide future treatment efforts within ED inpatient settings.

